# Mind-body practices for people living with HIV: a systematic scoping review

**DOI:** 10.1186/s12906-019-2502-z

**Published:** 2019-06-11

**Authors:** Maria Pilar Ramirez-Garcia, Marie-Pier Gagnon, Sébastien Colson, José Côté, Jorge Flores-Aranda, Myriam Dupont

**Affiliations:** 1 Faculty of Nursing, Université de Montréal, Montréal, Canada; 2 Research Center of the Centre Hospitalier de l’Université de Montréal, Montréal, Canada; 3 Faculty of Nursing, Université Laval, Québec, Canada; 4 Research Center of the Centre Hospitalier Universitaire, Québec, Canada; 5 Faculty of Medicine, Aix Marseille Université, Marseille, France; 6 University Institute on Addiction, Montreal-Island-South-Center Integrated University Health and Social Services Centre, Montréal, Canada; 7 Faculty of Medicine and Health Sciences, Université de Sherbrooke, Longueuil, Canada; 8 Library, Université Laval, Québec, Canada

**Keywords:** Mind-body practices, HIV, Tai chi, Yoga, Meditation, Relaxation, Progressive muscle relaxation, Hypnosis, Guided imagery, Autogenic training

## Abstract

**Background:**

Mind-body practices are frequently used by people living with HIV to reduce symptoms and improve wellbeing. These include Tai Chi, Qigong, yoga, meditation, and all types of relaxation. Although there is substantial research on the efficacy of mind-body practices in people living with HIV, there is no summary of the available evidence on these practices. The aim of this scoping review is to map available evidence of mind-body practices in people living with HIV.

**Methods:**

The Arksey and O’Malley (Int J Soc Res Methodol 8:19-32, 2005) methodological framework was used. A search of 16 peer-review and grey literature databases, websites, and relevant journals (1983–2015) was conducted. To identify relevant studies, two reviewers independently applied the inclusion criteria to all abstracts or full articles. Inclusion criteria were: participants were people living with HIV; the intervention was any mind-body practice; and the study design was any research study evaluating one or several of these practices. Data extraction and risk of bias assessment were performed by one reviewer and checked by a second, as needed, using the criteria that Cochrane Collaboration recommends for systematic reviews of interventions (Higgins and Green, Cochrane handbook for systematic reviews of intervention. 2011). A tabular and narrative synthesis was carried out for each mind-body practice.

**Results:**

One hundred thirty-six documents drawing on 84 studies met the inclusion criteria. The most widely studied mind-body practice was a combination of least three relaxation techniques (*n* = 20), followed in declining order by meditation (*n* = 17), progressive muscle relaxation (*n* = 10), yoga (*n* = 9) and hypnosis (*n* = 8). Slightly over half (47/84) of studies used a RCT design. The interventions were mainly (46/84) conducted in groups and most (51/84) included daily individual home practice. All but two studies were unblinded to participants.

**Conclusion:**

The amount of available research on mind-body practices varies by practice. Almost half of the studies in this review were at high risk of bias. However, mindfulness, a combination of least three relaxation techniques and cognitive behavioral strategies, and yoga show encouraging results in decreasing physical and psychological symptoms and improving quality of life and health in people living with HIV. More rigorous studies are necessary to confirm the results of Tai Chi, Qigong, and some relaxation techniques.

## Background

HIV is currently considered a chronic disease and people living with HIV are living longer [1, 2]. A recent study shows life expectancy in people undergoing antiretroviral therapy (ART) has increased by about 10 years. Twenty-year-olds starting ART in 2008–10 in Europe are expected to live to age 68, while their North Americas peers can hope to reach age 65 [3]. Despite the improved effectiveness of antiretroviral therapy, the quality of life of people living with HIV is strongly affected by the presence of one or more physical and psychological symptoms [4–6]. To reduce symptoms and treatment side effects and to improve general wellbeing, 55–60% of people living with HIV used complementary health approaches (CHA) [7–10]. They reported using CHA to round out conventional HIV care and antiretroviral treatment [7, 10, 11]. The National Center for Complementary and Integrative Health [12] defines CHA “as a group of health care approaches developed outside of mainstream Western or conventional medicine.” Most CHA fall into one of two subgroups: natural products or mind-body practices [12]. Some other CHA are traditional healers, Ayurvedic medicine, and traditional Chinese medicine [12].

Mind-body practices are frequently used by people living with HIV [7, 9, 13]. Mind-body practices users believe that the mind is an important components to wellbeing [14] and they use these practices to play an active role in their health management [2, 14]. Mind-body practices focus on the interactions between the mind, body, and behavior with the aim of affecting physical functioning and promoting health via the mind. Mind-body practices include Tai Chi, Qigong, yoga, meditation, and all types of relaxation (progressive muscle relaxation, hypnosis, guided imagery, breathing exercises, autogenic training, biofeedback and neurofeedback) [15]. Several systematic reviews show promising results of some mind-body practices in people with chronic disease. Two systematic reviews demonstrate that Tai Chi has beneficial effects on psychological wellbeing for various populations [16, 17]. Other systematic reviews showed that yoga improves exercise capacity and health-related quality of life in individuals with heart disease, stroke, and chronic obstructive pulmonary disease [18], and that relaxation reduces depression as compared to wait-list, no treatment, or minimal treatment in people diagnosed with depression or high-level symptoms of depression [19]. A RCT evaluating a 20-week intervention of supervised yoga practice found that systolic and diastolic blood pressures improved in people living with HIV at risk of developing cardiovascular disease [20]. Also, a pilot RCT found that daily meditation practice increased quality of life in people living with HIV [21].

Although there is substantial research on the effectiveness of mind-body practices on various populations, there is no summary of the available evidence on mind-body practices in people living with HIV. In addition, a preliminary review of published studies evaluating mind-body practices in people living with HIV found that the designs, interventions, and outcomes of these studies were very heterogeneous. Therefore, this review sought to map available evidence of mind-body practices for people living with HIV. Our study objectives were threefold: to provide an overview of the focus, quantity, and characteristics of existing research; to identify the types of available evidence of each mind-body practice in people living with HIV; and to highlight any gap in current knowledge in the goal of guiding future research.

## Methods

A systematic scoping review was carried out. A scoping review tends to address the broader topics when many different study designs might be applied [22]. Scoping reviews “aim to map rapidly the key concepts underpinning a research area and the main sources and types of evidence available, and can be undertaken as standalone projects in their own right, especially where an area is complex or has not been reviewed comprehensively before” [23].

To produce a thorough and systematic overview of existing research, we adopted the Arksey and O'Malley [22] methodological framework for scoping reviews. This review followed the five stages of this framework: 1) identifying the research question; 2) identifying relevant studies; 3) selecting the studies; 4) charting the data; and 5) collating, summarizing, and reporting results. For Arksey and O’Malley [22] study quality assessment is not part of the scoping review process. However, the lack of a process for assessing the quality of the scoping review is problematic, the danger being that the studies’ existence, rather than intrinsic quality, underpins conclusions [24]. Therefore, to identify the types of evidence available on mind-body practices in people living with HIV and to highlight any gaps in current research knowledge, we assessed the risks according to the criteria recommended in the *Cochrane Handbook for Systematic Reviews of Interventions* [25]. Furthermore, to operationalize the stages of selecting studies, charting data and summarizing results, we followed the Cochrane Collaboration’s recommendations for systematic reviews [25].

Before starting our scoping review, we wrote a review protocol describing the rationale and planned methods of the review. This protocol is available by contacting the corresponding author. The manuscript respects the Preferred Reporting Items for Systematic Reviews and Meta-Analyses (PRISMA) statement [26] because guidelines for reporting scoping review do not exist yet. However, some PRISMA items for systematic reviews are not appropriate for reporting a scoping review, particularly the items on the objectives, research questions and the conduct of a meta-analysis [27].

### Identifying the research question

Our research question asked “What is known from the existing literature about the effectiveness of mind-body practices for people living with HIV?” Based on the National Center for Complementary and Integrative Health [12] definition, mind-body practices are approaches that use the mind to affect physical functioning and promote health. Mind-body practices include Tai Chi and Qigong, yoga, meditation, and all types of relaxation (progressive muscle relaxation, hypnosis, guided imagery, deep-breathing exercises, autogenic training, biofeedback and neurofeedback). Although acupuncture is generally considered among mind-body practices, it was not included in this review because of its place in traditional Chinese medicine [15].

### Identifying relevant studies

To attempt to answer the research question, we identified primary studies and reviews in the literature, by searching electronic databases, websites, reference lists and key journals. A search strategy for electronic databases was developed according to the research question and key concepts. We used the following search terms: HIV OR AIDS AND mind-body therapy OR Alternative Medicine OR meditation OR yoga OR Tai Chi OR Qigong OR relaxation OR relaxation therapy OR biofeedback training OR biofeedback OR neurofeedback OR autogenic training OR deep-breathing OR guided imagery OR imagery OR progressive relaxation therapy OR muscle relaxation OR Jacobson progressive relaxation OR hypnosis OR hypnotherapy. Table 1 shows our Ovid search strategy. This was adapted for other databases, and details of those strategies may be obtained from the authors on request.

**Table 1:** Ovid MEDLINE®, In-Process & Other Non-Indexed Citations and OvidMEDLINE(R) 1946 to Present, search strategy

HIV/ OR	
exp HIV/	
exp HIV Infections/	
.ti,ab.	
AIDS	
HIV	
HIV-1*	
HIV1*	
HIV-2*	
HIV2*	
HTLV-III	
HTLV-3	
(human adj4 immun#deficiency virus*)	
(human immun#deficiency adj4 virus*)	
(human immun* and deficiency virus*)	
(acquired adj4 immun#deficiency syndrome*)	
(acquired immun#deficiency adj4 syndrome*)	
(acquired immun* and deficiency syndrome*)	
(human adj4 virus type III)	
(human adj4 virus type 3)	
lymphadenopathy-associated Virus*	
AND	
Mind-body therapy/OR	
exp Mind-Body Therapies/	
.sh.	
muscle relaxation	
.ti,ab.	
mindbody	
mind-body	
((alternative or complementary) adj4 (medicin* or therap*))	
meditation*	
yoga*	
yogic	
tai-chi	
taichi	
tai-ji	
taiji	
taijiquan	
relaxation*	
autorelaxation*	
biofeedback*	
myofeedback*	
neurofeedback*	
physiological feedback*	
autogenic training*	
deep-breath*	
breathing exercise*	
qigong	
qi gong	
chikung	
chi kung	
respiratory muscle* training*	
imagery	
imageries	
reverie	
visuali#ation*	
hypnos#s	
autohypnos#s	
hypnotherap*	
suggestion*	
autosuggestion*	
mesmerism*	

A total of 16 peer-review and grey literature databases from 1983 (when HIV was discovered) to October 2015 were searched. The peer-review database searched were: Cochrane Central Register of Controlled Trials (CCTR), Cochrane Database of Systematic Reviews, Cumulative Index to Nursing and Allied Health Literature (CINAHL), Database of Abstracts of Reviews of Effects (DARE), Health Technology Assessment, EMBASE, MEDLINE, PsycINFO and Web of Knowledge. The grey literature databases searched were: Google Scholar, Scirus, Archimède (Université Laval), Papyrus (Université de Montréal), Dissertations & Theses (ProQuest), the New York Academy of Medicine Grey Literature and OpenGrey. To identify ongoing clinical studies, we searched the following sites: ClinicalTrials, World Health Organization International Clinical Trials Registry Platform and Canadian trials. Our goal was to conduct a sensitive, rather than a specific, search of the literature. We enlisted the services of a qualified librarian (MD) to conduct the electronic database search. The last search was run on October 31, 2015.

This search was completed by examining the reference lists and citations of all included studies and by hand searching key journals, such as *BMC Complementary and Alternative Medicine*, *Journal of Evidence-Based Integrative Medicine*, *The Open Complementary Medicine Journal*, *Evidence-Based Complementary and Alternative Medicine*, *Complementary Therapies in Medicine*, *Complementary Therapies in Clinical Practice*, *Journal of Alternative and Complementary Medicine*, *Journal of Complementary & Integrative Medicine* and *European Journal of Integrative Medicine*. We considered publications in any language, as long as there was an English, French, or Spanish abstract.

### Selecting the studies

The search results were merged using reference management software to remove duplicate records of the same report. To identify relevant studies, we examined titles and abstracts using an eligibility checklist of our specific inclusion criteria. These were: participants were people living with HIV, the intervention was any mind-body practice, and the study design was any research study evaluating one or several of these practices. Following Levac, Colquhoun [28], two reviewers (PRG & SC) independently applied the inclusion criteria to all records. If the eligibility of a study was uncertain from the abstract, the full article was read. Discrepancies were resolved through discussion. All studies that met the inclusion criteria were eligible for the review.

### Charting the data

We developed an electronic data extraction form in Microsoft Excel with the following items: study ID, aims of the study, study design, total study duration, population, number of participants, setting, country, details and type of intervention (total number of intervention groups, specific intervention, length of intervention, number of contacts, provider and method of delivery), outcomes and time points, scales, and authors’ conclusions. Two reviewers (PRG & MPG) extracted data independently from 10 studies to determine if the approach to data extraction was consistent with the research question and purpose. The data were then extracted by one reviewer (PRG) and checked by another (MPG), as required. Discrepancies were resolved through discussion.

### Assessing risk of bias in included studies

Two reviewers (PRG & MPG) independently assessed the risk of bias for 10 of the included studies. Risk of bias was then assessed by one reviewer (PRG) and checked by another (MPG), as required. To asses randomized controlled trials (RCTs), we used the criteria recommended in the *Cochrane Handbook for Systematic Reviews of Interventions* [25] for six domains: selection bias (sequence generation and allocation concealment), performance bias (blinding of participants and health care providers), detection bias (blinding of outcome assessment), attrition bias, selective reporting and other potential sources of bias. Non-randomized controlled trials (NRCT) are defined here as any quantitative study estimating the effectiveness of an intervention that does not use randomization to allocate participants to comparison groups. This includes observational studies, with control before and after the study, interrupted-time-series studies and quasi-randomized studies. To assess the NRCT, we used the criteria recommended in the *Cochrane Handbook for Systematic Reviews of Interventions* [25] in six domains: selection bias (comparability of groups, confounding and adjustment), performance bias (blinding of participants and health care providers), detection bias (blinding of outcome assessment), attrition bias, selective reporting of outcomes and other potential sources of bias. Qualitative studies were evaluated using the criteria recommended by Spencer et al. [29] in two domains: approach (sampling strategy and sample selection criteria) and credibility (triangulation, respondent validation, plausibility, presentation of original data and link between data and conclusions).

### Collating, summarizing and reporting results

To answer the research question, we present the details of the included studies in tabular and narrative synthesis form, according to each mind-body practice. The tables include design, sample size, characteristics of intervention, and main results. We classified the studies by period of publication, country of origin, and mind-body practice to show the distribution of studies. For the narrative synthesis, we organized the studies by design, population characteristics, and simple size, and by the nature and characteristics of intervention. In accordance with Higgins and Green [25], the main results from NRCT and RCT are presented separately. The results from the qualitative data are used to explain and complement the results from the quantitative data. To present the evidence available for each mind-body practice and to highlight any gaps in current knowledge with a view to guiding future research, we interpreted the results with a consideration to the studies’ bias risk.

## Results

**Fig. 1:**
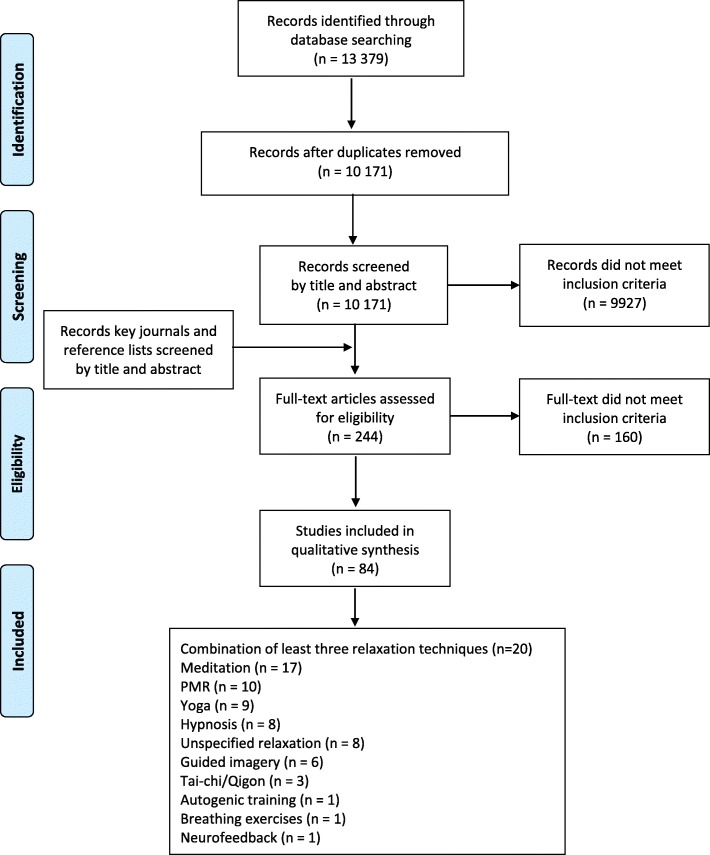
Study flow diagram

The search strategy identified 13,379 citations (Fig. 1). After removal of 3208 duplicates, 10,171 citations were screened by title and abstract. Of these, 9927 did not meet the criteria for inclusion. Therefore, 244 full text documents retrieved were screened for inclusion in the systematic scoping review. Finally, 136 documents met the inclusion criteria and were included in this systematic scoping review. These were 101 articles, 25 dissertations, and 10 conference abstracts, and they cover 84 studies (Fig. 1). Multiple documents on the same study were considered for the data extraction and are listed in the tables. The main study is indicated in **bold**. The most widely studied mind-body practice was a combination of least three relaxation techniques, followed by meditation, progressive muscle relaxation, yoga, hypnosis, guided imagery and Tai Chi/Qigong.

Three quarters of all studies (63/84) were performed in the United States, while seven were conducted in India, and two were carried out in Canada, China, the Netherlands and the United Kingdom. Iran, Japan, New Zealand, South Africa, Thailand and Zambia were the location for one study each. Seven studies were published before 1990. Thereafter, between 12 and 16 studies were published every 5 years, until 2010. Between 2011 and 2015, more than 20 published studies assessed mind-body practices in people living with HIV**.** Data from these studies show increasing interest in recent years in the HIV population for these types of practices.

We present below the details (design, sample size, intervention and main results by mind-body practice) of the studies included in this review.

### Tai Chi and Qigong

We identified only two published RCTs [30, 31] that assessed the effects of Tai Chi in people living with HIV. We also found one dissertation using a group pretest-posttest design to investigate the effects of Qigong on HIV infection [32] (Table 2). One RCT [30] collected qualitative data to explore participants’ perceptions of the efficacy of Tai Chi.

**Table 2:** Tai Chi and Qi gong included studies

Project identifier	Design	Sample size	Intervention/duration	Outcome/time
[32] USA	one group pretest-posttest design	26 QG	44 qigong g.s. of 2 h /12 weeks	Post-in ↓ depression ↓ anxiety ↑ T cell count
[30] USA	RCT + QA	13 TC 13 EX 12 CG	16 Tai Chi g.s. /8 weeks 16 exercise g.s. /8 weeks usual care	Post-int TC and EX ↑ physical function ↓ tension-anxiety ↑ QoL
[31, 33, 34] USA	RCT	65 CBR 62 TC 68 SP 57 CG	10 coping and relaxation g.s. /10 weeks 10 Tai Chi g.s. /10 weeks 10 spiritual growth g.s. /10 weeks usual care	Post-int & 6 months TC and CBR ↓ emotion-focused coping TC, CBR and SP ↑ lymphocyte proliferation

*QG* qigong, *g.s* group sessions, *Post-int* post intervention, *RCT* randomized control trial, *QA* qualitative approach, *TC* Tai Chi, *EX* exercise, *CG* control group, *QoL* quality of life, *CBR* cognitive-behavioral relaxation training, *SP* spiritual growth

The number of participants in two studies [30, 32] was close to 30, while McCain et al. (2008) had 200 in their study. Participants were people living with HIV [31, 32] or diagnosed with AIDS [30] without significant psychiatric or cognitive impairment [30, 31]. The interventions in all three of these studies were conducted in groups. One RCT study compared a short form of Tai Chi of eight movements (TC) with aerobic exercise (EX) and with usual care (CG) [30]. The other RCT compared Tai Chi to an intervention using a relaxation technique and coping strategies for stress management (CBR) with spiritual growth for enhancing exploration of the spiritual self (SP) and with usual care (CG) [31].

Although one RCT [30] was not powered to compare the two intervention groups (13 TC, 13 EX, and 12 CG), both intervention groups showed improvement at the post-intervention checkpoint in physical function, tension/anxiety, and quality of life compared to the control group. Qualitative data supported the benefits subjects experienced in the experimental groups on physical changes, mood, and social interaction [30]. The other RCT [31] showed a significant decline in usage of emotion-focused coping strategies between the pre-intervention and at the six-month follow-up visits in the TC and CBR groups. Furthermore, all intervention groups (CBR, TC and SP) had increased lymphocyte proliferative function as compared to the control group. The Qigong study [32] found significant differences between pre-intervention and post-intervention in depression and anxiety scores and in T-cell counts. The two RCTs studies were unblinded to participants, but one [31] was blinded to outcome assessors. Two studies [30, 32] presented high risk of attrition (Table 3).

**Table 3:** Risk of bias of included studies (Quantitative approaches)

Project identifier	Selection	Performance	Detection	Attrition	Reporting	Other
Tai Chi (*n* = 3)						
*NRCT*						
[32]	HR	HR	HR	HR	UR	LR
*RCT*						
[30]	UR	HR	UR	HR	LR	LR
[31]	LR	HR	LR	LR	LR	LR
Yoga (*n* = 9)						
*NRCT*						
[35]	HR	HR	HR	UR	LR	LR
[36]	HR	HR	HR	HR	LR	LR
[37]	HR	HR	HR	UR	LR	LR
*RCT*						
[38]	LR	HR	UR	HR	UR	LR
[20]	UR	HR	UR	UR	LR	LR
[39]	UR	HR	UR	LR	LR	LR
[40]	UR	HR	UR	LR	LR	LR
[41]	UR	HR	UR	LR	LR	LR
[42]	LR	HR	UR	HR	LR	LR
Meditation (*n* = 17)						
*NRCT*						
[43]	HR	HR	HR	UR	UR	UR
[44]	HR	HR	HR	HR	LR	LR
[45]	HR	HR	HR	UR	LR	LR
[46]	HR	HR	HR	UR	LR	LR
[47]	HR	HR	HR	LR	LR	LR
[48]	HR	HR	HR	UR	UR	UR
[49]	HR	HR	HR	HR	LR	LR
[50]	HR	HR	HR	LR	HR	LR
[51]	HR	HR	UR	UR	LR	LR
*RCT*						
[52]	UR	HR	UR	LR	LR	LR
[53]	UR	HR	UR	LR	LR	LR
[54]	LR	HR	UR	LR	LR	LR
[55]	UR	HR	LR	UR	LR	LR
[56]	LR	HR	HR	HR	LR	LR
[57]	UR	HR	UR	LR	LR	LR
[58]	LR	HR	UR	LR	LR	LR
PMR = (9)						
*NRCT*						
[59]	HR	HR	HR	HR	HR	LR
[60]	HR	HR	HR	LR	UR	LR
[61]	HR	HR	HR	UR	UR	UR
[62]	HR	HR	HR	HR	LR	UR
*RCT*						
[63]	UR	HR	UR	UR	LR	LR
[64]	UR	HR	UR	HR	LR	LR
[65]	UR	HR	UR	HR	LR	LR
[66]	UR	HR	UR	UR	HR	UR
[67]	UR	HR	UR	UR	LR	LR
Hypnosis (*n* = 8)						
*NRCT*						
[68]	HR	HR	HR	LR	LR	LR
[69]	HR	HR	HR	LR	LR	LR
[70]	HR	HR	HR	HR	LR	HR
[71]	HR	HR	HR	LR	LR	LR
[72]	HR	HR	UR	UR	LR	LR
[73]	LR	HR	LR	HR	LR	LR
*RCT*						
[74]	HR	HR	LR	HR	LR	HR
[75]	UR	HR	UR	UR	UR	UR
Guided imagery (*n* = 6)						
*NRCT*						
[76]	HR	HR	HR	HR	LR	LR
[77]	HR	HR	HR	LR	LR	LR
[78]	LR	HR	UR	UR	LR	LR
*RCT*						
[79]	UR	HR	UR	HR	LR	LR
[80]	LR	HR	UR	LR	LR	LR
[81]	LR	HR	UR	LR	LR	LR
Autogenic training (*n* = 1)						
*NRCT*						
[82]	HR	HR	HR	HR	LR	LR
Breathing exercise (*n* = 1)						
*NRCT*						
[83]	HR	HR	HR	LR	LR	LR
Neurofeedback (*n* = 1)						
*RCT*						
[84]	UR	HR	UR	LR	LR	LR
Combination of least three relaxation techniques (*n* = 20)						
*NRCT*						
[85]	HR	HR	HR	LR	LR	LR
[86]	HR	HR	HR	HR	LR	LR
[87]	HR	HR	HR	LR	LR	LR
[88]	HR	HR	UR	LR	LR	LR
[89]	HR	HR	HR	HR	LR	LR
[90]	LR	UR	UR	HR	LR	LR
*RCT*						
[91]	UR	HR	UR	HR	LR	LR
[92]	UR	UR	UR	UR	UR	UR
[93]	UR	HR	UR	HR	LR	LR
[94]	UR	HR	UR	LR	LR	LR
[95]	LR	HR	UR	HR	LR	LR
[96]	UR	HR	UR	HR	LR	LR
[97]	UR	HR	UR	LR	LR	LR
[98]	UR	HR	UR	HR	LR	LR
[99]	UR	HR	UR	HR	LR	LR
[100]	LR	HR	LR	LR	LR	LR
[101]	LR	LR	UR	LR	LR	LR
[102]	UR	HR	UR	HR	LR	LR
[103]	UR	HR	UR	HR	LR	LR
[104]	LR	LR	UR	LR	LR	LR
Unspecified relaxation (*n* = 8)						
*NRCT*						
[105]	HR	HR	HR	HR	LR	LR
[106]	UR	UR	UR	UR	LR	LR
*RCT*						
[107]	UR	HR	UR	UR	UR	LR
[108]	UR	HR	UR	HR	LR	LR
[109]	UR	HR	UR	UR	LR	LR
[110]	UR	HR	UR	HR	LR	LR
[111]	UR	HR	UR	LR	LR	LR
[112]	HR	HR	UR	HR	LR	LR

### Yoga

We identified 11 papers, including 8 articles and 3 conference abstracts, that described 9 studies assessing the effects of yoga on people living with HIV [20, 35–42]. Five studies used a RCT design [20, 38–41], one had a RCT pilot design [42], and three [35–37] a one-group pretest-posttest design (Table 4). Two studies [36, 38] collected qualitative data to explore the participants’ perspective on the yoga’s effect on their wellbeing.

**Table 4:** Yoga included studies

Project identifier	Design	Sample size	Intervention/duration	Outcome/time
[35] USA	one group pretest-posttest design	48 BMSG	10 yoga, nutrition, hardiness, alternatives therapies g.s. /10 weeks	Post-int
[36] USA	one group pretest-posttest design + QA	4 YG	16 Iyengar yoga g.s. /8 weeks	Post-int ↓ pain ↓ anxiety
[37] India	one group pretest-posttest design	96 YGNA	Twice daily yoga, hydrotherapy, diet therapy and sun bath /1 to 180 days – (average- 4 weeks)	Post-int ↑ CD4 counts
[38] Canada	RCT + QA	20 YG	27 yogic breathing and meditation g.s. /14 weeks	Post-int ↑ well-being
		27 CG	usual care	
[20, 113] USA	RCT	29 YG	18 Ashtanga Vinyasa yoga i.s. + 22 Ashtanga Vinyasa yoga g.s. /20 weeks	Post-int ↓ trans fat intake ↓ blood pressure
		21 CG	usual care	
[39] conference abstract India	RCT	YG 35	90 yoga s. /12 weeks	Post-int ↓ distress
		CG 35	usual care	
[40, 114] Zambia	RCT	adolescents		Post-int ↓ emotional symptom ↑ CD4 counts
		12 YGPS	20 yoga g.s. and group discussion /10 weeks	
		11 PS	20 group discussion /10 weeks	
		11 CG	usual care	
[41] India	RCT	31 YG	12 sudarshan kriya yoga g.s. of 2 h /12 weeks	Post-int ↑ QoL
		30 CG	usual care	
[42] USA	pilot RCT	12 YG	16 yoga g.s. /8 weeks	Post-int & 4 months Feasible and acceptable ↓ distress ↓ stress
		12 CG	usual care	

*BMSG* behavioral medicine support group, *g.s* group sessions, *Post-int*: post-intervention, *QA* qualitative approach, *YG* yoga, *RCT* randomized control trial, *CG* control group, *i.s* individual sessions, *YGPS* yoga and peer support, *PS* pair support, *YGNA* yoga and naturopathy interventions, *QoL* quality of life

Three studies had fewer than 15 participants per group [36, 40, 42]. The number of participants per group in the other studies ranged from 20 [20, 38] to 96 [37]. Most were conducted on samples of people living with HIV [35] in stable condition and without significant psychiatric or cognitive impairment [20, 38] or cardiac problems [20, 41]. One study was conducted with participants with pain or anxiety [36] and another [42] with people living with HIV who used crack cocaine. One study was conducted with adolescents, aged between 11 and 16 years, who were aware of their HIV status and had been receiving antiretroviral treatment for at least 1 year [40]. Four out of five RCTs compared yoga intervention to usual care. One RCT [40] compared a yoga and peer-support intervention to peer-support intervention only and to usual care. Six studies [35, 36, 38, 40–42] evaluated yoga in groups. The intensity and duration of interventions varied between 10 and 90 sessions within a 4–26-week period; the most frequent duration was, however, between 8 and 12 weeks.

One-group studies showed post-intervention effects on pain, anxiety [36], and CD4 counts [37]. RCTs showed that yoga could decrease psychological distress [40, 42], and increase quality of life [38, 41] and CD4 counts [40] immediately after the intervention. Qualitative data suggest that participants perceived yoga as a way to gain awareness and constructively manage the physical and emotional challenges related to their HIV [36, 38]. One RCT [20] showed a decrease in trans-fat consumption and in participants’ blood pressure after the group’s yoga intervention, as compared to the control group. Only one pilot RCT [42] showed that the effects of yoga on psychological distress were maintained 2 months after the intervention. All RCTs were unblinded to participants. They did not give information on blinding of outcome assessors. Three studies [36, 38, 42] presented high risk of attrition (Table 3).

### Meditation

We identified 23 papers (17 articles, 5 dissertations and 1 conference abstract) reporting on 17 studies that assessed any form of meditation in people living with HIV. Seven studies used a RCT design [52–58], one had a pilot RCT design [21], six used one-group pretest-posttest design [43–48], two had a non-equivalent untreated control group with pretest and posttest design [49, 51] while one action research design was built with mixed methods [50]. Five studies collected qualitative data to explore the perceived benefit from meditation to the participants (Table 5).

**Table 5:** Meditation included studies

Project identifier	Design	Sample size	Intervention/duration	Outcome/time
[43] USA	one group pretest-posttest design	175 MD	10 meditation i.s. + mantra’s repetition daily /10 weeks	Post-int ↓ distress
[44] USA	one group pretest-posttest pilot design	5 MBCT	8 mindfulness-based stress reduction and cognitive therapy g.s and 1 mindfulness-based stress reduction and cognitive therapy i.s. + daily practice /8 weeks	Post-int & 4 month ↓ depressive symptoms ↑ dispositional mindfulness ↑ QoL
[45, 115, 116] USA	one group pretest-posttest pilot design + QA	adolescents 59 (11HIV+) MBSR	9 body scan, mindfulness meditation, yoga g.s of 2 h + daily practice /8 weeks	Post-int Feasible and acceptable ↓ symptoms (hostility and discomfort)
[46] USA	one group pretest-posttest pilot design + QA	11 MCPA	5 noticing positive events, capitalizing, gratitude, mindfulness, positive reappraisal, personal strengths, attainable goals, kindness, i.s. + daily home practice /6 weeks	Post-int Feasible and acceptable ↑ positive affect ↓ negative affect
[47] USA	one group pretest-posttest pilot design + QA	9 MABT	8 massage, interceptive training, mindful body awareness i.s. of 1 h30 + home practice /8 weeks	Post-int Feasible and acceptable
[48] Conference abstract UK	one group pretest-posttest pilot design + QA	12 CFT	8 attention, emotional regulation, mindfulness practice g.s. of 2 h /8 weeks	Post-int Feasible and acceptable
[49] USA	untreated control group with pretest and posttest design	24 MBSR	8 body scan, mindfulness meditation, hatha yoga g.s. of 2 h30 + daily home practice /8 weeks	Post-int & 5 months ↓ mood disturbance ↓ stress ↑ NK cell number and activity
		10 CG	usual care	
[50] Thailand	action research: mixed method	16 PSM	8 mindfulness meditation, emotional reflective techniques s. + daily home practice /8 weeks	Post-int ↓ suffering
[51, 117] USA	untreated control group with pretest and posttest design + QA	21 3S+	12 cognitive-behavioral spirituality focused psychotherapy, mindfulness training i.s. + daily mindfulness home practice /12 weeks	Post-int ↓ impulsivity ↑ spirituality
		17 CG	usual care	
[52] USA	RCT	10 ST	4 support group g.s. of 1 h30 /4 weeks	
		10 CBST	4 Benson’s relaxation exercises, cognitive-behavioral stress training g.s. of 1 h30 + twice daily home practice/ 4 weeks	
		10 WL	wait-list	
[53] USA	RCT	13 MT	1 meditation g.s. of 90 min + daily meditation home practice /4 weeks	Post-int & 2 months To MT + MSSG: ↑ QoL
		13 MSSG	20 massage i.s. of 30 min /4 weeks	
		16 MT + MSSG	1 meditation g.s of 90 min + daily meditation home practice + and 20 massage i.s of 30 min /4 weeks	
		16 Control	usual care	
[54, 118] USA	RCT	46 MT	6 mantra’s repetition g.s. of 90 min and group discussion + daily practice /10 weeks	Post-int & 22 weeks ↓ anger ↑ spiritual wellbeing
		47 ACG	6 videotapes on HIV-topics g.s. of 90 min and group discussion /10 weeks	
[55, 119] USA	RCT	33 MBSR	8 body scan, mindfulness meditation, yoga g.s. of 2 h + a day long retreat (6 h) + daily practice /10 weeks	Post-int = CD4 counts
		15 CG	1 day MBSR information	
[56, 120] Iran	RCT	120 MBSR	8 body scan, mindfulness meditation, yoga g.s. of 2 h + 1 day of retreat (6 h) + daily practice /8 weeks	Post-int & 12 months ↓ physical symptoms
		125 ESC	2 HIV education g.s of 2 h	
[57] USA	RCT	40 MBSR	8 body scan, mindfulness meditation, yoga g.s. of 2 h30 + 1 day of retreat (6 h) + daily practice /8 weeks	Post-int ↓ ARV side effects
		36 CG	wait-list	
[58] Canada	RCT	78 MBSR	8 body scan, mindfulness meditation, yoga g.s. of 3 h + 1 day of retreat (6 h) + daily practice /8 weeks	Post-int & 6 months ↓ avoidance ↑ positive affect ↑ mindfulness
		39 CG	usual care	
[21] USA	pilot RCT	11 TM	14 transcendental meditation s. + daily home practice /24 weeks	Post-int Feasible and acceptable ↑ HRQoL
		11 HE	14 healthy eating education s.	

*MD* meditation, *i.s*. individual sessions, *Post-int* post intervention, *MBCT* mindfulness-based cognitive therapy, *g.s* group sessions, *QoL* quality of life, *QA* qualitative approach, *MBSR* mindfulness-based stress reduction program, *RCT* randomized controlled trial, *QE* qualitative evaluation, *MCPA* multiple-component positive affect, *MABT* mindful awareness in body-oriented therapy, *CFT* compassion-focused therapy, 3-S+ spiritual self-schema therapy, *PSM* palliative-suffering model, *TM* transcendental meditation, *HE* healthy eating education, *HRQoL* health related quality of life, *ST* support training, *SIT* stress inoculation training, *WL* wait list, *MT* mantra, *MSSG* massage, *MT + MSSG* mantra + massage, *ACG* attention control group, *CG* control group, *ESC* education and support condition

Most studies were conducted on general samples of people living with HIV [21, 46, 47, 49, 50, 52, 54, 56] with moderate distress [44, 55] or ARV side effects [57]. One study was conducted on drug users living with HIV who were enrolled in an inner-city methadone maintenance program; [51] another on gay men living with HIV [58], and a third on near end-of-life AIDS patients [53]. The participants of one study were adolescents (aged 13–21), a third of whom was living with HIV [45]. One study included people who had never tested, who were HIV-, HIV+ or who were living with AIDS [43]. Most studies excluded people with cognitive impairment, psychosis, or substance use. The number of participants ranged from 5 [44] to 245 [56]. Seven studies had fewer than 15 participants per group [21, 44, 46–48, 52, 53]. The number of participants per group in other studies ranged from 15 [55] to 125 [56]. Six studies assessed the effect of a mindfulness-based stress reduction (MBSR) intervention [45, 49, 55–58]. The MBSR group intervention consisted of eight weekly sessions of 2–3-h each, a daylong retreat, and daily home practice. MBSR meditation practices include body-scan meditation, yoga postures practiced with mindful awareness of the body, and sitting meditation with mindfulness of breath, thoughts, and emotions. The other forms of meditation assessed were mantra repetition [43, 54]. Metta meditation [53], transcendental meditation [21] and any form of mindfulness training combined with other cognitive, emotional, or spiritual techniques [44, 46–48, 50, 51]. A study [52] assessed an intervention with two components: a cognitive-behavioral stress training and Benson relaxation response. The duration of these interventions varied between 4 [52, 53] and 24 weeks [21], but the most frequent duration was between 8 and 12 weeks [44, 47, 50, 51, 54]. All the interventions included daily home practice.

One group pretest-posttest design found that mantra repetition reduced distress in people who had never tested for HIV, HIV- people and HIV+ people [43]. Two NRCTs studies found that MBSR reduced hostility, discomfort symptoms [45], mood disturbance and stress [49]. Qualitative data suggested that MBSR helped participants feel calmer, happier and more able to manage their anger and conflicts [45]. Other very small NCRTs (*n* = 5) found that mindfulness training combined with other cognitive, emotional, or spiritual techniques reduced depression symptoms [44], negative affects [46], and impulsivity [51], and it increase quality of life [44]. Participants in an intervention using any form of mindfulness training and massage explained that they had learned self-care skills based on self-awareness, and that the intervention had had a positive effect on their symptom management and on facing up to the challenges of living with HIV [47]. Similarly, participants in an intervention combining mindfulness meditation with emotional reflective techniques stated that the intervention helped them understand and accept the nature of life, manage their behaviors, and initiate self-healing to alleviate their suffering [50].

Three RCTs studies found that MBSR reduced physical symptoms [56] and ARV side effects [57], and increased positive affect at post-intervention and at 6 months [58]. One RCT founds that mantra repetition reduce distress and increase spiritual wellbeing immediately after the intervention and after 3 months [54]. A small RCT (*n* = 42) [53] found that meditation combined with massage was more effective in increasing the quality of life of people living with HIV approaching end of life than meditation or massage alone. Finally, a pilot RCT found that daily practice of transcendental meditation could be effective to increase quality of life in people living with HIV [21]. All studies were unblinded to participants and only two studies [21, 55] were blinded to outcome assessors. Three studies [44, 49, 56] presented high risk of attrition (Table 3).

### Relaxation

A total of 97 documents were reviewed. These consisted of 73 articles, 19 dissertations, and 5 conference abstracts drawing on 55 studies that assessed one or more relaxation techniques in people living with HIV. Ten of these studies assessed progressive muscle relaxation, eight examined hypnosis, and six looked at guided imagery. Only one study was found for breathing exercises, autogenic training, and neurofeedback techniques. Additionally, 20 studies assessed a combination of at least 3 relaxation techniques with or without other components. Finally, eight studies assessed a relaxation technique combined with cognitive behavioral therapy, without however specifying the relaxation technique used. The following sections present the main findings of these studies.

#### Progressive muscle relaxation

We identified 16 papers, including 12 articles, 3 dissertations and 1 conference abstract, discussing progressive muscle relaxation effects in people living with HIV, as presented in 10 different studies [59–67, 121–127]. Five studies used a RCT design [63–67], three used one-group pretest-posttest design [59–61], and one had a phenomenological design [121]. One study [62] began by randomizing participants into three groups. However, since almost half of the subjects assigned to group intervention refused to participate, they were reassigned to the comparison group (Table 6).

**Table 6:** Progressive muscle relaxation included studies

Project identifier	Design	Sample size	Intervention/duration	Outcome/time
[59, 122] India	one group with multiple assessment pretest- posttest	6 CBT	20 psychoeducation, activity scheduling, cognitive restructuring, behavioral counseling, progressive muscular relaxation with biofeedback i.s. of 1 h	Post-int ↓ symptoms ↓ depression ↓ anxiety
[60] USA	one group pretest-posttest design	5 CBT + PMR	12 adherence training, behavioral activation, cognitive restructuring, problem solving, progressive muscle relaxation and diaphragmatic breathing i.s. / 12 weeks	Post-int ↑ adherence ↓ depression
[61] India	one group pretest-posttest pilot design	30 JPMR	10 Jacobson’s progressive muscle relaxation s. /10 weeks	Post-int ↓ anxiety ↓ depression
[121] USA	phenomenology	24 PMRT + GI	1 progressive muscle relaxation and guided imagery i.s or g.s. of 30 min	Post-int feeling satisfied, refreshed and peaceful
				relief muscle tension and stress
[62] Chine	Three group pretest-posttest design	10 CBT	12 cognitive restructuring, behavior change strategies, assertiveness skills, progressive muscle relaxation, home practice g.s. of 2 h /12 weeks	Post-int & 6 month ↑ mood ↑ QoL ↑ CD4
		10 PSC	12 discussion by leaders, feelings, problems, fears and hopes g.s of 2 h /12 weeks	
		26 CG	usual care	
[63, 123–127] USA	RCT	14 CBSM	20 cognitive restructuring, assertiveness skills, behavior change strategies, stress information, progressive muscle relaxation, imagery component g.s. of 45 min to 1 h30 + home practice /10 weeks	Post-int CBSM et AE ↑ CD4 counts ↓ depression
		19 AE	30 aerobic exercise g.s. of 45 min	
		15 CG	usual care	
[64] USA	RCT	27 CB/PMR	8 cognitive and behavioral strategies, social support, progressive muscle relaxation g.s of 1 h30 + home practice /8 weeks	Post-int CB/PMR and SSG ↓ depression ↓ hostility ↓ somatization SSG ↓ anxiety
		14 SSG	8 social support group discussion g.s. of 1 h30 /8 weeks	
		27 CI	crisis intervention	
[65] Netherlands	RCT	8 CBT	15 cognitive restructuring, behavior change strategies, assertiveness skills, stress management techniques and progressive muscle relaxation exercise of Bernstein & Borkovec g.s. of 2 h30 + 1 day 8-h + relaxation home practice /15 weeks	Post-int CBT and ET ↓ depression ↑ mood
		6 ET	15 group discussion by leaders and progressive muscle relaxation exercise of Bernstein & Borkovec g.s. of 2 h30 + 1 day 8-h + relaxation home practice /15 weeks	
		6 WL	wait-lits	
[66] JAPAN	RCT	6 PMR + AT	progressive muscle relaxation and autogenic training /12 weeks	Post-int PMR + AT and PSY: ↓ anxiety ↓ depression ↓ confusion ↓ fatigue
		6 PSY	supportive psychotherapy/ 12 weeks	
		7 WL	waiting list	
[67] USA	adolescents	12 Massage	24 massage therapy i.s. of 20 min /12 weeks	Post-int Massage ↓ depression ↑ immune function
	RCT	12 PMR	24 progressive relaxation routine Jacobson i.s. of 20 min /12 weeks	

*CBT* cognitive-behavioral group psychotherapy, *i.s* individual sessions, *Post-int* post intervention, *PSC* peer/support counseling, *CG* control group, *g.s* group sessions, *QoL* quality of life, *CBT + PMR* cognitive behavioral therapy and progressive muscle relaxation, *JPMR* Jacobson’s progressive muscle relaxation, *s* sessions, *PMRT + GI* progressive muscle relaxation training and guided imagery, *RCT* randomized control trial, *CBSM* cognitive behavioral stress management, *AE* aerobic exercise, *CG* control group, *CB/PMR* cognitive and behavioral strategies, social support, progressive muscle relaxation, *SSG* support social group, *CI* crisis intervention, *ET* experiential group psychotherapy, *WL* waiting-list control, *PMR + AT* progressive muscle relaxation and autogenic training, *PSY* psychotherapy

Six studies had fewer than 15 participants per group [59, 60, 62, 65–67], while 4 had between 15 and 30 participants in each group [61, 63, 64, 121]. Four studies recruited people living with HIV who showed a stable condition [61, 66, 67, 121] and two studies recruited people with symptomatic HIV infection [59, 62]. The population of three studies consisted of peopling living with HIV who presented problems related to anxiety or depression [59, 62]. One study enrolled healthy homosexual men who were unaware of their HIV-1 antibody status [63]. In general, participants with any history of drug or alcohol abuse [63, 65] or with any current and major psychiatric disorder [59, 60, 62, 65] were excluded. Four studies offered group interventions [62–65], while three had individual interventions, and [59, 60, 67] one offered either individual or group intervention [121]. Two studies did not specify their offering [61, 66]. The number of meetings varied between a one-time 30-min meeting [121] and nearly twenty 20–90-min meetings [59, 63, 67] over a period of 8 [64] to 20 weeks [59]. Six studies combined progressive muscle relaxation with different cognitive behavioral strategies [59, 60, 62–65] and offered daily relaxation practice at home [62–65]. Another study combined progressive muscle relaxation with autogenic training [66], while another combined this type of relaxation with guided imagery [121]. Two other studies assessed progressive muscle relaxation as the only intervention applied [61, 67].

Combining progressive muscle relaxation with cognitive behavioral strategies, three very small studies with a one-group pretest-posttest design [59, 60, 62] suggest their interventions could reduce physical symptoms, as well as anxiety and depression, and increase patient compliance and quality of life. Participants from a phenomenological study reported felling refreshed and peaceful after 30-min progressive muscle relaxation and guided imagery practice. They also noted release of their physical manifestations of worry, frustration, anxiety and stress [121]. Furthermore, Bommareddi and Valsaraj [61] showed in India that just a 10-week practice of progressive muscle relaxation reduced anxiety and depression in people living with HIV.

Antoni and his colleagues [63] found that an intervention using progressive muscle relaxation and cognitive behavioral strategies reduces the distress and immune system deterioration in gay men associated with notification of the serological status. Moreover, the post-notification distress and immunologic values were strongly associated with self-reports of daily relaxation practice at home. Two RCTs combining progressive muscle relaxation with cognitive behavioral strategies [64, 65] showed the beneficial effects of these interventions on symptoms of depression in people living with HIV. A small three-arm RCT [66] compared an intervention combining progressive muscle relaxation and autogenic training (PMR + AT) to psychotherapy (PSY) and to a wait list group (WL) for 12 weeks. The results indicated the effectiveness of relaxation techniques on anxiety, depression, confusion and fatigue, but no significant differences in the effectiveness of relaxation techniques compared to psychotherapy. Finally, comparing massage to progressive muscle relaxation over a 12-week period in a group of adolescents living with HIV (*n* = 24), a small RCT [67] found beneficial results on depression and on immune system function among the adolescents receiving massage but not those in the relaxation group. All RCTs studies were unblinded to participants and they did not give information on blinding of outcome assessors. Four studies [59, 62, 64, 65] were at high risk of attrition

Antoni and his colleagues [63] found that an intervention using progressive muscle relaxation and cognitive behavioral strategies reduces the distress and immune system deterioration in gay men associated with notification of the serological status. Moreover, the post-notification distress and immunologic values were strongly associated with self-reports of daily relaxation practice at home. Two RCTs combining progressive muscle relaxation with cognitive behavioral strategies [64, 65] showed the beneficial effects of these interventions on symptoms of depression in people living with HIV. A small three-arm RCT [66] compared an intervention combining progressive muscle relaxation and autogenic training (PMR + AT) to psychotherapy (PSY) and to a wait list group (WL) for 12 weeks. The results indicated the effectiveness of relaxation techniques on anxiety, depression, confusion and fatigue, but no significant differences in the effectiveness of relaxation techniques compared to psychotherapy. Finally, comparing massage to progressive muscle relaxation over a 12-week period in a group of adolescents living with HIV (*n* = 24), a small RCT [67] found beneficial results on depression and on immune system function among the adolescents receiving massage but not those in the relaxation group. All RCTs studies were unblinded to participants and they did not give information on blinding of outcome assessors. Four studies [59, 62, 64, 65] were at high risk of attrition (Table 3).

#### Hypnosis

We identified 10 documents (5 articles, 4 dissertations, and 1 conference abstract), which drew on 8 studies assessing hypnosis in people living with HIV [68–75]. Only two studies used a RCT design [74, 75]. Two other studies [72, 73] used an untreated control group with pretest and posttest design, and the last four [68–71] used a one-group pretest-posttest design (Table 7). (Table 7).

**Table 7:** Hypnosis included studies

Project identifier	Design	Sample size	Intervention/duration	Outcomes/time
[68, 128] USA	one group pretest-posttest design	5 HYP	10 deep breathing, hypnotic induction, pain relief and sleep suggestions i.s. + twice daily hypnosis home practice /10 weeks	Post-int ↓ pain ↓ pain medication
[69] New Zealand	one group pretest-posttest design	3 HYP	6 guided muscle relaxation, visualization, imagery and suggestions to control the itch i.s. + 30 min hypnosis home practice /6 weeks	Post-int ↓ itch
[70] India	one group pretest-posttest design	20 HYP	9 progressive muscular relaxation, ego strengthening, guided imagery, visualization, and sensory imagery conditioning techniques g.s. of 2 h + hypnosis home practice /9 weeks	Post-int ↑ coping
[71**,** 129] USA	one group pretest-posttest design	41 HYP	3 hypnosis i.s of 70 min + self-hypnosis practice with specific target goals /3 weeks	Post-int & 7 weeks ↓ pain ↑ QoL
[72] USA	untreated control group with pretest and posttest design	14 HYP	6 inner guide techniques, the shamanic journey technique, mental imagery involving the immune system i.s. of 20 min + daily self-hypnosis home practice /6 weeks	Post-int ↓ anxiety ↑ CD4/CD8 ratio
		9 CG	usual care	
[73] USA	untreated control group with pretest and posttest design	16 HYP	1 hypnosis and imagery training to relief distress, to address immune functioning and to impart a sense of self-efficacy and control i.s. + daily hypnosis home practice /12 weeks	Post-int
		17 WL	wait list	
[74] USA	RCT	19 HYP	3 stress discussion and hypnosis i.s. of 45 min + daily hypnosis home practice /1 week	Post-int
		15 CON	3 stress discussion i.s. of 45 min	
		9 CG	usual care	
[75] India	RCT	90 HYP	16 progressive muscular relaxation, ego strengthening, changing of negative mood set to positive mood set and boosting up of immune system g.s. of 90 min + self-hypnosis home practice/ 16 weeks	Post-int & 6 months ↑ vitality
		90 CG	usual care	

HYP: hypnosis; i.s.: individual sessions; Post-int: post intervention; g.s.: group sessions; QoL: quality of life; CG: control group; WL: wait list; RCT: randomized controlled trial; CON: counseling;

Most of the studies had a small sample size. Only four included more than 15 participants per group [70, 71, 73, 75]. Some studies recruited HIV+ gay men [72, 74] or stable HIV people [70, 73]. Two studies enrolled people living with HIV and pain [68, 71], while two others recruited people living with HIV and dermatitis [75] or pruritus [69]. These studies excluded participants with disabling neurological impairment, drug addiction, or alcoholism [72, 74], as well as those with any other acute or chronic illnesses [70, 71, 73] or severe depression [75]. Six studies offered individual hypnosis [68, 69, 71–74] ranging from 1 [73] to 10 sessions [68] that lasted 20–70 min. Two studies [70, 75] consisted of nine 2-h group interventions [70] or sixteen 1.5-h group interventions [75]. All the interventions include of daily self-hypnosis practice at home.

Results of three small NRCTs studies suggested hypnosis had beneficial effects on pain [68], itching [69], and anxiety [72]. Two studies with a one group pretest-posttest design and with samples of more than 15 participants found beneficial results of their intervention on coping [70], pain, and quality of life [71]. The final study we identified assessed individual hypnosis over a three-week period found beneficial results on pain and quality of life 1 month after the end of the intervention [71]. One RCT [75], which assessed sixteen 1.5-h sessions of group hypnosis found positive results on the vitality of people living with HIV and dermatitis. Another RCT [74] assessing a one-week individual hypnosis intervention does not show significant results. All studies were unblinded to participants and only one [74] was blinded to outcome assessors. Three studies [70, 73, 74] were rated high risk of attrition.

#### Guided imagery

We found 10 documents (5 articles and 5 dissertations) drawing on six studies that assessed guided imagery in people living with HIV. Two studies [79, 80] used a RCT design, and another [81] had a pilot RCT design. One study [78] used an untreated control group and two others [76, 77] had a one-group pretest-posttest design (Table 8).

**Table 8:** Guided imagery included studies

Project identifier	Design	Sample size	Intervention/duration	Outcome/time
[76] USA	one group pretest-posttest design	7 GIR	6 guided imagery and relaxation g.s of 1 h30 + home practice /6 weeks	Post-int ↑ self-control ↓ anxiety
[77, 130] USA	one group pretest-post-test design	20 VT	1 visualization training with verbal suggestion and visualization instructions i.s. of 30 min + daily home practice /5 days	Post-int ↑ white blood cell count
[78] USA	untreated control group with pretest and posttest design	30 MSG	1 slow stroke back massage i.s. of 30 min	Post-int MSG and GI ↓ pain ↓ anxiety ↓ depression ↑ self-esteem
		30 GI	1 autogenic induction and guided imagery i.s. of 30 min	
[79] USA	RCT	11 PST	8 problem solving training g.s. of 1 h30 + homework /8 weeks	Post-int. & 3 moths RGIT ↓ mood disturbance ↓ hopelessness
		12 RGIT	8 relaxation and guided imagery training g.s. of 1 h30 + twice daily home practice /8 week	
		13 CG	control group	
[80, 131, 132] USA	RCT	23 GI	1 relaxation induction and guided imagery i.s. of 22 min + daily practice /6 weeks	Post-int GI ↓ depression ↓ fatigue PMR ↓ depression ↑ CD4+
		22 PMR	Progressive muscle relaxation home practice /6 weeks	
		24 CG	usual care	
[81, 133] USA	pilot RCT	30 CSMT	1 stress, cognitive appraisal process, coping strategies, guided imagery i.s. of 1 h30 + home practice /4 weeks	Post-int ↑ stress management knowledge
		30 WL	wait list	

*GIR* guided imagery and relaxation, *g.s* group sessions, *Post-int* post intervention, *VT* visualization training, *MSG* massage, *GI* guided imagery, *i.s* individual sessions, *RCT* randomized controlled trial, *PST* problem solving training, *RGIT* relaxation and guided imagery training, *PMR* progressive muscle relaxation, *CG* control group, *CSMT* computerized stress management training, *WL* wait list

Two studies [76, 79] had fewer than 15 participants per group and the other studies varied between 20 [77, 80] and 30 participants [78, 81]. They were composed of men [79], women [81], or both genders living with HIV [76, 80]. Two studies included patients with a variety of diagnoses, such as cancer, AIDS, viral infections or post-surgical recovery. Participants with cognitive impairment or drug or alcohol addictions were excluded. Four studies [77, 78, 80, 81] offered guided imagery as an individual intervention. One included a single 30-min session of guided imagery [78] and the other three [77, 80, 81] offered one training session followed by daily practice over 5 days to 6 weeks. The other two studies offered 1.5-h sessions of guided imagery in small groups once a week, over a period of six [76] to 8 weeks [79] with daily practice at home.

A very small NRCT (*n* = 7) suggested the existence of the beneficial effects of guided imagery on anxiety and self-control [76]. Two NRCTs studies with sample sizes of more than 15 participants found beneficial effects on anxiety, depression, pain, self-esteem [78] and white blood cell counts [77]. RCTs results show decreased symptoms of depression, fatigue [80], and mood disturbance [79] after relaxation induction and guided imagery was practiced once or twice per day. Results on mood disturbance were maintained a month after the end of the intervention [79]. All studies were unblinded to participants and they did not give information on blinding of outcome assessors. Two studies [76, 79] were deemed at a high risk of attrition.

#### Autogenic training, breathing, and neurofeedback

We found four documents reporting on three studies that assessed autogenic relaxation technique [82], neurofeedback [84], and breathing exercises [83] in people living with HIV. Neurofeedback was assessed using an RCT design in one of these studies [84] and the other two [82, 83] used a one-group pretest-posttest design (Table 9). Two of the studies assessed the combination of autogenic training exercises [82] or breathing exercises [83] with physical, mental, and spiritual activities over a seven to eight week period. Neurofeedback was compared to home cranial electrotherapy and to the combination of these two interventions over a 16-week period.

**Table 9:** Autogenic training, breathing and neurofeedback included studies

Project identifier	Design	Sample size	Intervention/duration	Outcome/time
[82] UK	one group pretest-post-test design	50 AT	7 diet, nutrition, supplements, exercise, safer sex, recreational drugs, spiritual experiences, autogenic training g.s. of 2 h + autogenic exercises home practice /7 weeks	Post-int & 8 months ↑ quality of life
[83] USA	one group pretest-post-test pilot design	13 SCGI	8 spirituality, mental and physical health, personal goals, relaxation breathing exercise g.s. of 1 h15 /8 weeks	Post-int ↑ positive spiritual coping ↓ depression
[84**,** 134] USA	RCT	10 NFB	32 neurofeedback i.s. of 20 min /16 weeks	Post-int NFB and NFB + HDCE ↓ symptoms ↑ CD4+
		10 HDCE	20 min daily cranial electrotherapy home practice /16 weeks	
		10 NFB+ HDCE	32 neurofeedback i.s. of 20 min + 20 min daily cranial electrotherapy home practice /16 weeks	
		10 WL	wait list	

*AT* autogenic training, *g.s* group sessions, *Post-int* post intervention, *SCGI* spiritual coping group intervention, *RCT* randomized control trial, *NFB* neurofeedback, *HDCE* home daily cranial electrotherapy, *NFB + HDCE* neurofeedback and home daily cranial electrotherapy, *WL* wait list, *i.s* individual sessions

A study assessing autogenic training in groups over 7 weeks found beneficial results for quality of life in people living with HIV/AIDS both immediately and 8 months after the intervention [82]. A small study (*n* = 13) assessing a spiritual coping group intervention, which includes breathing exercises, showed an increase in the use of positive spiritual coping and a decrease in symptoms of depression after the intervention in a group of adults living with HIV [83]. Finally, neurofeedback alone or in combination with daily home cranial electrotherapy seemed to decrease symptoms and increase CD4 levels in people living with HIV with CD4 counts between 150 and 650/cc [84]. The RCT was unblinded to participants and this did not give information on blinding of outcome assessors. One study [82] was deemed at high risk of attrition.

#### Combination of at least three relaxation techniques

We identified 47 documents (39 articles, 6 dissertations, and 2 conference abstracts) drawing on 20 studies that assessed a combination of at least 3 relaxation techniques with or without other components in people living with HIV [85–104] (Table 10). Most studies (13/20) used an RCT design [91–93, 95–104]. One [94] used a pilot RCT design and collect qualitative data to describe participants’ perceptions about the efficacy of the intervention. Four studies [87–90] used an untreated control group with pretest and posttest design and two studies [85, 86] had a one-group pretest-posttest design with a very small size sample.

**Table 10:** Combination of at least three relaxation techniques included studies

Project identifier	Design	Sample size	Intervention/duration	Outcome/time
[85] USA	case series (one group pretest-post-test design)	2 SMT	13 stress management training, progressive relaxation, autogenic training, and/or guided imagery augmented with biofeedback i.s. of 1 h30 + relaxation home practice /13 weeks	Post-int ↓ anxiety ↓ physiological stress symptoms
[86] USA	case series (one group pretest-post-test design)	3 B/AB/ PMR/GI	8 biofeedback of abdominal breathing, progressive muscle relaxation, guided imagery i.s. of 1 h30 + relaxation home practice /8 weeks	Post-int ↓ symptom severity ↓ stress ↓ mood disturbance
[87] USA	untreated control group with pretest and post-test design	1 B/PMR/AT	20 biofeedback, progressive muscle relaxation, autogenic phrases i.s. of 1 h + daily relaxation home practice /10 weeks	Post-int ↓ muscle tension ↓ stress ↑ lymphocyte immune system functioning
		1 CG	usual care	
[88] USA	untreated control group with pretest and post-test design	30 SMT	6 cognitive restructuring, active coping skills, progressive muscle relaxation, yoga form, thematic imagery, meditation g.s. of 1 h + daily home practice /6 weeks	Post-int ↑ well being
		15 CG	usual care	
[89, 135] USA	untreated control group with pretest and post-test design	60 CBT	12 pain management, cognitive reconceptualization, restructuring, diversion and behavioral activation, problem solving, progressive muscle relaxation, guided imagery and breathing g.s. of 1 h30 /12 weeks	Post-int & 6 months ↓ pain
		30 CG	usual care	
[90, 136] USA	untreated control group with pretest and post-test design	196 RES led	10 CBSM+ g.s. of 2 h + 6 healthy life styles (adherence, nutrition, physical activity, sexual risk and alcohol and drug use) g.s of 2 h by research staff /16 weeks	Post-int To RES-led and CHC-led: ↑ nutrition ↑ physical activity ↓ sexual intercourse ↓ tobacco use
		168 CHC led	10 CBSM+ g.s. of 2 h + 6 healthy life styles (adherence, nutrition, physical activity, sexual risk and alcohol and drug use) g.s of 2 h by community health center /16 weeks	
		64 obs	only observation	
[91] USA	RCT	13 B/AT/ GI/H	8 thermal biofeedback, autogenic training, guided imagery, hypnosis g.s. of 1 h30 + daily home practice /8 weeks	Post-int ↓ symptoms ↑ vigor ↑ hardiness
		13 WL	wait list	
[92] USA	RCT	*N* = 10 ? BSM	20 progressive muscle relaxation, biofeedback assisted relaxation, self-hypnosis, meditation i.s. of 1 h + home practice /10 weeks	Post-int ↓ anxiety ↓ mood disturbance ↑ T cell count ↑ self-esteem
		? CG	usual care	
[93, 137–140] USA	RCT	22 CBSM	10 stress and emotion, rational thought replacement, coping skills, assertiveness training, anger management, identification social support, relaxation techniques (progressive muscle relaxation, autogenic training, meditation, breathing exercise), g.s. of 2 h15 + twice daily relaxation home practice /10 weeks	Post-int ↓ dysphoria ↓ anxiety ↓ HSV-2 antibody titers ↑ coping skills ↑ social support
		18 CG	usual care	
[94, 141] USA	Pilot RCT + QA	34 PSMP	7 symptoms, medication, communication with caregivers, cognitive-behavioral strategies, muscle relaxation, guided imagery, visualization, prayer or meditation g.s. of 2 h30 + homework /7 weeks	Post-int ↑ self-efficacy ↓ symptoms
		37 CG	usual care	
[95] USA	RCT	18 RT	7 breathing, autogenic relaxation, progressive muscle relaxation, imagery techniques s. of 2 h and 15 min + daily home practice /12 week	Post-int ↑ health behavior ↑ CD4+ cells ↓ viral load
		11 WL	wait list	
[96] USA	RCT	8 Massage	12 massage i.s. of 45 min / 12 weeks	Post-int To massage + ex and massage + REL ↓ medical care utilization To massage + REL ↑ health perceptions
		8 Massage +EX	12 massage i.s. of 45 min + 12 aerobic exercise i.s. of 20 min + 12 aerobic exercise i.s. of 45 min /12 weeks	
		8 Massage + REL	12 massage i.s. of 45 min + 12 biofeedback, diaphragmatic breathing and autogenic relaxation i.s. of 45 min	
		7 CG	usual care	
[97] USA	RCT	20 SM	14 cognitive behavioral management skills, anxiety, anger and depression management, coping skills, abdominal breathing, progressive muscle relaxation and autogenic training i.s. of 90 min /7 weeks	Post-int ↓ mood disturbance (anger, confusion, tension, depression and fatigue) ↑ coping ↑ health perceptions
		20 WL	usual care	
[98, 142–150] Dissertation USA	RCT	62 CBSM	10 stress and emotion, cognitive restructuring, coping skills, assertiveness training, anger management, identification and using social support, relaxation techniques (progressive muscle relaxation, imagery, autogenic training, meditation, breathing exercise) g.s. of 2 h15 + twice daily relaxation home practice /10 weeks	Post-int ↓ depression ↓ mood disturbance ↓ dysfunctional attitudes ↓ stress ↓ norepinephrine ↓ cortisol ↑ coping strategies ↑ free testosterone ↑ social support
		38 WL	wait list	
[99] USA	RCT	59 CBSM	8 cognitive restructuring techniques, active coping skills, breathing, progressive muscle relaxation, yoga form stretching, guided imagery g.s. of 1 h30 + daily relaxation practice /8 weeks	Post-int To CBSM: ↑ quality of life (emotional well-being)
		43 SSG 36 WL	8 emotional communication, problem solving, cognitive reframing, and empowerment g.s. of 1 h30 /8 weeks	
[100**,** 151] USA	RCT	76 CBSM +MAT	10 stress management and relaxation practice (progressive muscle relaxation, imagery, autogenic training, meditation, breathing exercise) g.s. of 2 h15 + twice daily relaxation home practice /10 weeks and 3 adherence information i.s. /9 month	Post-int & 15 months ↓ depressed mood ↓ denial coping ↓ viral load
		54 MAT	3 i.s of information adherence/ 9 month	
[101**,** 152] USA	RCT	58 AC + RR	12 acupuncture and relaxation techniques (breathing, mental repetition, autogenic, guided body scan, visualization and guided imagery) i.s. of 45 to 60 min + twice daily relaxation home practice /12 weeks	Post-int ↑ QoL
		61 AC	12 acupuncture and soft music i.s. of 45 to 60 min	
[102**,** 153] USA	RCT	44 CBSM	10 stress management and relaxation practice g.s. of 2 h15 relaxation techniques (progressive muscle relaxation, imagery, autogenic training, meditation, breathing exercise) + twice daily relaxation home practice /10 weeks	Post-int & 9 months ↑ affect ↑ social support ↓ stress ↓ cervical neoplasia
		26 WL	wait list	
[103, 154–161] USA	RCT	212 CBSM+	10 CBSM g.s. of 2 h (stress and emotions, coping skills, assertiveness training, anger management, social support, progressive muscle relaxation, diaphragmatic breathing, guided imagery) + relaxation home practice/ 10 weeks	Post-int & 6 and 12 months ↑ self-efficacy ↑ QoL ↑ cognitive functioning ↓ depression & anxiety by women who
		239 III	10 information intervention with videotape i.s. of 2 h/ 10 weeks	
[104] USA	RCT	31 AC + RR	12 acupuncture and relaxation techniques (breathing, mental repetition, autogenic self-hypnosis, guided body scan, visualization and guided imagery) i.s of 30 min + relaxation home practice /8 week	Post-int ↓ symptoms (loose stools, diarrhoea, gas/bloating) (The interventions of acupuncture and the RR were more effective when used in combination than when used alone)
		27 AC + HE	12 acupuncture and management of HIV symptoms information i.s of 30 min /8 weeks	
		27 SAC + RR	12 sham acupuncture and and relaxation techniques (breathing, mental repetition, autogenic self-hyponis, guided body scan, visualization and guided imagery) i.s. of 30 min + relaxation home practice /8 week	
		30 SAC + HE	12 sham acupuncture and management of HIV symptoms `information i.s. of 30 min /8 weeks	

*STM* Stress management training, *i.s* individual sessions, *Post-int* post intervention, *B/AB/PMR/GI* biofeedback of abdominal breathing, progressive muscle relaxation, guided imagery, *B/PMR/AT* biofeedback, progressive muscle relaxation and autogenic training, *SMT* stress management training, *CG* control group, *g.s* group sessions, *CBT* cognitive-behavioral therapy, *RES led* research led, *CHC-led* community health center led, *obs* observation only group, *B/AT/GI/H* biofeedback, autogenic training, guided imagery and hypnosis, *WL* wait list, *BSM* behavioral stress management, *CBSM* cognitive stress management training, *QA* qualitative approach, *PSMP* positive self-management program, *RT* relaxation training, *massage + EX* massage and aerobic exercise, *massage + REL* massage and relaxation, *SM* stress management, *SSG* social support group, *CBSM+MAT* cognitive behavioral stress management and medication adherence training, *MAT* medication adherence training, *AC* acupuncture, *RR* relaxation, *HE* health education, *SAC* sham acupuncture

Seven studies [85–87, 91, 92, 95, 96] had less than 15 participants per group. The size of the other studies varied between 40 [93, 97] and over 400 participants [90, 103]. Most recruited stable seropositive people [85–88, 90–93, 95–99, 103]. Six other studies [89, 94, 100, 101, 104] recruited symptomatic seropositive people or women with a cervical lesion [102]. Most studies excluded participants with cognitive impairment, psychiatric disorder, or drug or alcohol addictions. Seven studies [85–87, 91, 92, 95, 96] assessed a combination of at least three relaxation techniques, including biofeedback, breathing, progressive muscle relaxation, autogenic training, guided imagery and hypnosis. Most of these interventions (5/7) were offered individually and included home practice (6/7). They were divided into 7 to 20 sessions, lasting between 1 and 2 h, for between 8 and 13 weeks. Seven studies [90, 93, 98–100, 102, 103] assessed the same cognitive-behavioral stress management intervention (CBSM) intervention. In this intervention, participants attended 10 weekly 2.25-h group sessions (broken down into 90 min of cognitive behavioral (CB) training and 45 min of relaxation) and were instructed to practice relaxation exercises twice a day between sessions. The relaxation exercises included progressive muscle relaxation, autogenic training, meditation and deep breathing. Four other studies [88, 89, 94, 97] assessed a combination of relaxation techniques, yoga exercises, and/or meditation with cognitive-behavioral strategies. Three of these offered group interventions [88, 89, 94] and the fourth had individual sessions. Interventions consisted of six to twelve 1–2.5 h sessions over 6–12 weeks. Two interventions included the relaxation practice at home. Two studies [101, 104] assessed a set of relaxation techniques used in combination with acupuncture. These individually offered interventions included 7–20 sessions over 6–16 weeks with relaxation exercises at home.

Three NRCTs, which had two to three participants [85–87] and assessed a combination of at least three relaxation techniques without other components, found beneficial results on anxiety, mood disturbance, stress, symptom severity and lymphocytes count. Four small RCTs [91, 92, 95, 96] also examining this type of intervention showed beneficial effects post intervention on general symptoms, energy, confidence, anxiety, distress, health-related behaviors, use of health services, viral load and CD4 counts. However, these studies were small with fewer than 15 participants per group.

One NRCT [90] found that CBSM intervention in women living with HIV was associated with improvement in behavioral outcomes, and these outcomes were comparable between groups led by research and community health centers. Six RCTs [93, 98–100, 102, 103] that assessed CBSM showed beneficial results on anxiety, distress, coping abilities, social support, stress, cortisol and testosterone levels, cognitive function, perceived self-efficacy, cervical neoplasia, quality of life and viral load. One of these studies [93] found that the number of weeks an individual practiced relaxation at least once was significantly correlated with changes in depression and anxiety. Three of these studies showed positive results of the interventions over the long-term (6–15 months) on perceived self-efficacy [103], stress, cervical neoplasia [102] and viral load [100].

Two pretest and posttest studies [88, 89] with an untreated control group found that a combination of relaxation techniques and meditation exercises with cognitive-behavioral strategies improved wellbeing [88] and decreased pain [89]. Increased weekly practice was found to be significantly related to increased positive impact on stress, higher quality of life, more frequent use of problem-focused coping, less avoidant thinking and uncertainty [88]. Effects on pain were long-term (more than 6 months) [89]. Two RCTs [94, 97] that assessed a combination of relaxation techniques and meditation exercises with cognitive-behavioral strategies showed beneficial results on symptoms, mood disturbance, perceived self-efficacy, coping and health perceptions. Finally, two RCTs that assessed a combination of relaxation techniques, such as breathing, autogenic training, body-scan exercise and guided imagery with acupuncture found beneficial results on quality of life [101] and general symptoms [104]. All but two studies [101, 104] were unblinded to participants; one study [100] was unblinded to participants, but blinded to outcome assessors. Eleven studies [86, 89–91, 93, 95, 96, 98, 99, 102, 103] were rated at high risk of attrition (Table 3).

#### Unspecified relaxation

We found 10 documents (9 articles and 1 dissertation) on 8 studies [105–112] that assessed cognitive-behavioral strategies with a relaxation technique without indicating the type of relaxation technique used (Table 11). They consisted of six RCTs [107–112], one untreated control group with pretest and posttest design [106], and one group pretest-posttest design [105].

**Table 11:** Unspecified relaxation included studies

Project identifier	Design	Sample size	Intervention/duration	Outcome/time
[105] USA	case series (one group pretest-post-test design)	4 CBT-AD	8 behavioral activation, cognitive restructuring, problem solving, adherence tools, relaxation i.s.	Post-int ↓ depression ↑ adherence
[106] USA	untreated control group with pretest and post-test design	*N* = 50 ? SMG	10 stress management techniques, relaxation training, problem-solving skills and effective coping styles g.s. of 2 h /10 weeks	Post-int ↑ mood ↑ coping
		? CG	usual care	
[107] USA	RCT	*N* = 64 ? SMG	8 systematic relaxation, health habit change, skills for managing stress g.s. of 2 h /8 weeks	Post-int ↓ sexual partners
		? WL	wait-list	
[108] USA	RCT	32 C	1 tailored counseling i.s	3 months To C + SPT + R: ↓ anxiety ↓ psychiatric symptoms
		31 C + V + R	3 counseling, information video about HIV and relaxation (abdominal breathing, imaging i.s. of 45 min /3 weeks	
		40 C + SPT + R	6 tailored counseling, cognitive behavioral treatment for depression, stress and anxiety (education, personal control, training to manage challenging situations) and systematic relaxation i.s. of 1 h / 6 weeks	
[109**,** 162] South-Africa	RCT	14 EG	8 cognitive-behavioral therapy, aerobic exercise, biofeedback-assisted relaxation i.s. of 1 h30 + relaxation home practice /8 weeks	Post-int ↓ depression ↑ mood ↑ lymphocyte counts
		12 CG	8 individual counseling (psychological and medical aspects of the AIDS) i.s. of 1 h30 /8 weeks	
[110, 163] USA	RCT	46 CET	10 stress management, coping strategies, social support, relaxation g.s. of 1 h30 on 10 weeks + 6 boosters stress management sessions /12 months	Post-int & 6 and 12 months ↓ stress ↓ burnout ↓ anxiety ↑ coping self-efficacy
		44 HIV-I	10 HIV information g.s. of 1 h30 on 10 weeks + 6 boosters information sessions /12 months	
		31 WL	wait-list	
[111] Netherland	RCT	44 GI	17 stress management, coping skills, social support, relaxation, written HIV information g.s. of 2 h30 on 16 weeks + 5 stress management, coping skills, social support, relaxation g.s. /9 months	Post-int ↑ coping
		41 EC	written hiv information	
[112] China	RCT	6 CBP	7 coping skills, social support, cognitive restructuring, behavior change strategies, relaxation, g.s. of 2 h + daily relaxation home practice/ 7 weeks	Post-int ↑ mood ↑ QoL
		7 WL	wait list	

*CBT-AD* cognitive behavioral technique and adherence tools, *i.s* individual sessions, *Post-int* post intervention, *SMG* stress management group, *g.s* group sessions, *CG* control group, *WL* wait list, *C* counseling, *C + V + R* counseling, video and relaxation, *C + SPT + R* counseling, stress prevention training and relaxation, *EG* experimental group, *CET* coping effectiveness training, *HIV-I* information about HIV, *GI* group intervention, *EC* educational control, *CBP* cognitive behavioral program

Three studies had fewer than 15 participants per group [105, 109, 112]. The sample size of the other studies varied between 50 [106] and 120 [110]. Participants were heterosexual [112] and homosexual or bisexual men [106] living with HIV [107, 109, 111]. Two studies [105, 110] recruited homosexual or bisexual men living with HIV and depression. One study recruited adults with perceived risk for HIV infection [108]. Most studies excluded participants with cognitive impairment, major medical or mental illness, and current alcohol or drug use. Most interventions (5/8) were offered to groups [106, 107, 110–112]. They consisted of between 3 and 22 meetings varying in length between 45-min and 2.5 h and held between 3 and 16 weeks. Two interventions [110, 111] added 5 or 6 boost sessions over a 9–12-month period.

Two NRCTs studies [105, 106] showed positive results on mood, coping, and adherence. Six RCTs [107–112] found beneficial results on reducing the number of sexual partners, anxiety, depression, mood and stress, and improving lymphocyte count and quality of life. Two of the studies did not describe attrition [107, 109] and the rest, which presented attrition rates between 14 and 20%, did not consider losses in their analysis. All studies were unblinded to participants and did not give any information on blinding of outcome assessors. Four studies [105, 108, 110, 112] were rated as a high risk of attrition.

## Discussion

The amount of research on mind-body practices in people living with HIV over 30 years varies widely depending on the practice. Data from these studies show an increasing research interest over the last 5 years for these practices (particularly meditation and yoga) in the HIV population. Research on hypnosis also seems to have increased slightly in recent years, while the number of studies on progressive muscle relaxation and guided imagery appear to be decreasing. However, research on a combination of least three relaxation techniques seems to remain stable, with three or four studies for every five-year period since 1990.

About two-thirds of the studies examining a combination of at least three relaxation techniques, yoga, and Tai Chi/Qigong, and half of the studies on progressive muscle relaxation, meditation, and guided imagery used a RCT design. Most studies were conducted on general samples of people living with HIV and excluded participants with any history of drug or alcohol abuse, current psychiatric major disorders, or cognitive impairment. Half of the studies on hypnosis and on progressive muscle relaxation and some of the studies on yoga, meditation or on a combination of at least three relaxation techniques recruited participants living with HIV who have depression, anxiety, pain or other symptoms. Three studies—on mindfulness [45], on yoga [40], and on progressive muscle relaxation [67]—were conducted with adolescents to assess the effects of these techniques on their psychological wellbeing. Two studies—on yoga and on meditation—were conducted on drug users to assess the effects of these interventions on quality of life [42] and on abstinence [51]. Two studies from the 1990s recruited people with perceived risk for HIV [108] or who were unaware of their HIV antibody status [63] in the goal of examining relaxation’s effectiveness in reducing emotional distress after testing. Interventions were mainly conducted in groups with the exception of hypnosis, guided imagery, and neurofeedback, which were performed individually. All studies on hypnosis and most studies on meditation, guided imagery, and a combination of least three relaxation techniques consisted of individual daily practice at home. The duration of these interventions varied between a few hours and 1 year. The most frequent duration for yoga, meditation, and a combination of at least three relaxation techniques was 8 weeks. The most frequent length of progressive muscle relaxation interventions was 12 weeks, and Tai Chi/Qigong interventions lasted between 8 and 12 weeks. The duration of hypnosis and guided imagery was extremely variable.

In this systematic scoping review, the results of three rigorous RCTs show that meditation and, specifically mindfulness-based stress reduction (MBSR) decrease physical symptoms and ARV side effects [56, 57] and improve the psychological state and mindfulness of people living with HIV [58]. These results are confirmed by a systematic review [164] of MBSR for people living with HIV. Indeed, we found that the studies reviewed offer preliminary support for positive effects of MBSR on health and emotional wellbeing. A systematic review [165] of meditation in chronic disease found that most studies (24/45) were based on MBSR and this intervention improves symptoms of anxiety and depression and decreases symptoms in chronic diseases. An overview of systematic reviews and meta-analyses of RCTs using MBSR-based interventions [166] found that MBSR improves symptoms of depression, anxiety and stress, as well as the quality of life and physical functioning in individuals with various conditions, as compared to wait-list control and usual care groups. A well designed RCT study included in this scoping review [54] suggests that repeating a mantra for 5 weeks can decrease psychological distress in people with HIV. More studies are needed to confirm these findings on mantras and identify the effects of other types of meditation, such as transcendental meditation, in people living with HIV.

Two well-designed RCTs [94, 97] assessing a combination of at least three relaxation techniques, with meditation exercises, and cognitive-behavioral strategies show beneficial results on symptoms, mood disturbance, cognitive functioning, and health perceptions. A rigorous RCT [100] show that a cognitive-behavioral stress management (CBSM) program, a combination of relaxation techniques (progressive muscle relaxation, autogenic training and deep breathing) and cognitive behavioral training, had positive effects on depression and viral load. Four others RCTs with some major methodological limitations [93, 98, 102, 103] found that a CBSM program seemed decrease anxiety and depression and to increase the cognitive functioning, quality of life, and social support of people living with HIV. Studies with other populations show results similar to CBSM on physical symptoms [167], psychological symptoms [168, 169], and quality of life [169]. However, it should be noted that this type of intervention is very complex and therefore not very accessible. Furthermore, much of the intervention is based on the practice of these relaxation techniques. In this regard, two well-designed RCTs [101, 104] examining the effects of adding a combination of relaxation techniques (deep breathing, autogenic training, body-scan exercise and guided imagery) to usual acupuncture found that this combination had more effects on decresing gastrointestinal symptoms and increasing quality of life than acupuncture alone. Four small RCTs [91, 92, 95, 96] assessing a combination of at least three different relaxation techniques suggest positive effects on symptoms, anxiety, distress, health behavior, viral load and CD4 count in people living with HIV. However, further rigorous research with larger sample sizes are necessary to assess the effect on people living with HIV of the combination of at least three relaxation techniques without other strategies.

Due to the major methodological limitations of these studies, there is little evidence of the effectiveness of each relaxation technique used separately. Some RCT included in this scoping review (but holding high risk of bias in some domains) found that progressive muscle relaxation [64, 65, 126] improved the psychological state of people living with HIV. The results of two NRCT studies [68, 71] suggest that hypnosis decreased pain and improved quality of life in this population. The findings of two NRCT [76, 78] and two small RCTs [79, 80] suggest that guided imagery had some effect on mood improvement. And an NRCT study on autogenic training [82] and a RCT on neurofeedback [84] show positive effects on the quality of life and the symptoms of people living with HIV. These results are consistent with those provided by some systematic reviews of progressive muscle relaxation [19, 170, 171], hypnosis [172], guided imagery [173], autogenic training [174], and neurofeedback [175] in a variety of populations. Except neurofeedback, which requires specialized equipment and professionals, all the relaxation techniques studied are very accessible, inexpensive, and easy to practice following brief training. Moreover, these relaxation practices involve low emotional and physical risk and allow patients to take a more active role in their treatment. Further research is necessary to prouve the effects of these relaxation techniques used separately by people living with HIV.

A rigorously led RCT [20] found that systolic and diastolic blood pressures improved more in yoga group after 20 weeks of supervised Asthange Vinyasa practice and home practice than in the standard of care group of HIV-infected adults with displaying cardiovascular disease risk factors. A systematic review [176] concluded that yoga appears to be promising in modifying risk factors for cardiovascular disease and metabolic syndrome in adults. Furthermore, results from two one-group studies [36, 37] and from four RCT with some important methodological limitations suggest that this practice could be used to decrease psychological distress [40, 42], increase quality of life [38, 41], and improve the immune system [40] in people living with HIV. Similary, a systematic review of a randomized control trial on the effects of yoga on mood and stress-reduction measures in diverse populations [177] suggests that yoga leads to better regulation of the sympathetic nervous system and the hypothalamic-pituitary-adrenal system, as well as to decrease in symptoms of depression and anxiety. However, more rigorous RCTs are needed to confirm these findings on the emotional and immunological status and quality of life and to identify the effects of long-term yoga practice in people living with HIV.

There is little evidence of the effectiveness of Tai Chi and Qigong in people living with HIV. A well-designed RCT included in this systematic scoping review [31] found that a 10-weeks group Tai Chi intervention for decreased the use of emotion-focused coping strategies and increase lymphocyte proliferation function in people living with HIV. One study with a one group pretest-posttest design suggested that Qigong may improve emotional and immunological status in this population [32]. These results seem to be consistent with those obtained from various populations living with chronic disease. In fact, results from systematic reviews show that Tai Chi improves physical performance in people living with cancer, osteoarthritis, heart failure and chronic obstructive pulmonary disease [178], as well as with symptoms of depression and anxiety in elderly and other populations living with chronic disease [16]. The practice of Qigong also seems to have a positive effect on the severity of depressive symptoms in people living with chronic disease [179]. Additionally, some studies reported improvements in a number of immune-related blood markers among Qigong and Tai Chi practitioners [180], but more studies are needed to confirm these results on the immune system [181]. For people living with HIV, more rigorous RCT are needed to confirm the results of Tai Chi and Qigong on physical, emotional, and immunological status as well as on quality of life.

### Strengths and limitations

The Arksey & O’Malley framework [22] provides an appropriate methodological foundation for scoping reviews (Levac & al., 2010). The combination of this method with Cochrane recommendations for systematic reviews of interventions [25] allowed us to consider the risk of bias in the interpretation of study results and to specify the steps of research, identification, data extraction and synthesis. The combination of these methods led us to call the review “a systematic scoping review.” An additional strength of this type of review is to present the results according to the type of mind-body practice. This strategy makes it possible to better understand evidence-based results available for each type of mind-body practice and to identify the lack of existing knowledge for each practice.

Nonetheless, this scoping systematic review also has some limitations. This review includes studies published only until October 2015. Although a thorough search strategy was used, there may still be gaps from unpublished data mainly from studies from 90s. Some trials did not provide descriptions of the nature of the mind-body practices tested. The limitations of evidence-based results from the studies included in this review must also be added. Only slightly over half of the studies (47/84) used an RCT design. All but two studies were unblinded to participants and only five used assessors blinded to treatment allocation. Another important problem is the high attrition rate (33/84) and a success rate for interventions that fell below 60% for several studies. Furthermore, most studies did not address incomplete data adequately and excluded dropouts from the final analysis. Finally, some studies were too small to draw any firm conclusions. Risk of bias, however, tended to be lower in the studies published more recently, as compared to older studies.

## Conclusions

### Implications for clinical practice

In our systematic scoping review, we showed that some mind-body practices have encouraging results in improving physical and psychological wellbeing and health in people living with HIV. The practices for which there is better evidence are mindfulness, a combination of at least three relaxation techniques with cognitive behavioral strategies, and yoga. An 8-weeks of mindfulness-based stress reduction (MBSR) program could be suggested to decrease physical symptoms and ARV side effects, and improve the psychological state of people living with HIV. A combination of at least three relaxation techniques and cognitive behavioral strategies could be used to decrease physical and psychological symptoms, and increase quality of life and health in this population. A combinaison of deep breathing, autogenic training, body scan, guided imagery, and acupuncture could be suggested to people living with HIV and gastrointestinal symptoms. Asthange Vinyasa practice could be recommended to lower blood pressure in pre-hypertensive people living with HIV who also display mild-to-moderate cardiovascular disease risk factors. Tai Chi, Qigong, and relaxation techniques used separately may also contribute to the improvement of the physical and psychological condition of people living with HIV, but more rigorous studies are necessary to confirm these results in people living with HIV.

### Implications for research

Future clinical trials should focus on rigorous study design to minimize bias risks and enhance validity. Although it is difficult with this type of intervention to blind participants, the outcome assessors should be blinded to treatment allocation. All randomized participants should be included in their analysis and intention-to-treat analysis should be used to avoid overoptimistic estimates of the efficacy of interventions [182]. Adhering more closely to CONSORT [183] guidelines for good reporting of clinical trials would considerably help increase the amount of available data. Follow-up on the evaluation of mind-body practices would be important to identify required dosage and modality of interventions for people living with HIV and to accurately determine the contribution of these practices to the health of this population.

## Abbreviations

AIDS: Acquired immune deficiency syndrome
ART: Antiretroviral therapy
CBSM: Cognitive-behavioral stress management intervention
CHA: Complementary health approaches
HIV: Human immunodeficiency virus
MBSR: Mindfulness-based stress reduction
NRCT: Non-randomized controlled trials
RCT: Randomized controlled trials
